# Movements of Magnetite-Encapsulated Graphene Particles at Air–Water Interface and Their Cell Growths under Dynamic Magnetic Field

**DOI:** 10.3390/nano13202806

**Published:** 2023-10-22

**Authors:** Jia Ji Lee, Misganu Chewaka Fite, Toyoko Imae, Poh Foong Lee

**Affiliations:** 1 Lee Kong Chien Faculty of Engineering & Science, Universiti Tunku Abdul Rahman, Jalan Sungai Long, Bandar Sungai Long, Cheras, Kajang 43000, Malaysia; jiajilee@hotmail.com; 2 Graduate Institute of Applied Science and Technology, National Taiwan University of Science and Technology, 43 Section 4, Keelung Road, Taipei 10607, Taiwan; d10619803@mail.ntust.edu.tw

**Keywords:** translational movement, self-rotational movement, magnetite-encapsulated graphene particle, air–water interface, external magnetic field, electromagnet system, cell growth induction

## Abstract

The motion of magnetic particles under magnetic fields is an object to be solved in association with basic and practical phenomena. Movement phenomena of magnetite-encapsulated graphene particles at air–water interfaces were evaluated by manufacturing a feedback control system of the magnetic field to cause the motion of particles due to magnetic torque. A homogeneous magnetic field was generated using two pairs of electromagnets located perpendicular to each other, which were connected to an electronic switch. The system influenced the translational movement and the self-rotational speed of magnetic particles located at a center on the surface of fluid media in a continuous duty cycle. Operating the particle at a remote control in the same duty cycle at the air–water surface, the short and elongated magnetic particles successfully rotated. In addition, the rotational speed of the curved particle was slower than that of the elongated particle. The results indicate that the translational and self-rotational movements of magnetite-encapsulated graphene particles at the air–water interface under the external magnetic field are size- and shape-dependent for the speed and the direction. A short magnetic particle was used as a target particle to rotate on cancer cell lines, aiming to study the advantage of this method to induce the growth of HeLa cells. It was monitored for up to 4 days with and without magnetic particles by checking the viability and morphology of cells before and after the electromagnetic treatment. As an outcome, the movement of magnetic particles reduced the number of biological cells, at least on HeLa cells, but it was inactive on the viability of HeLa cells.

## 1. Introduction

Graphitic materials incorporated magnetic nanoparticles with magnetic nature have gained much interest and have been used for various applications including magnetic resonance imaging [1], magnetic bioseparation [2], drug delivery [3], withdrawal of water pollutants [4], removal of dyes [5], adsorption of heavy metal ions and radionuclides [6], electrode [7], sensing [8], and electromagnetic heating [9] in the different areas such as biomedical research [1], environmental science and catalysis [10], magnetic energy storage [11], and magnetic fluidics [12]. Moreover, the effect of magnetism on magnet organisms with organic magnetic materials in their body is not necessarily elucidated. Thus, the influence on organisms (especially on cells) by the movement of magnetic materials under a magnet is also a target issue.

External (either static or dynamic field type) magnetic field is a powerful tool to exert a force on materials with magnetic moment localized in its region. When magnetic materials are exposed to the external field, materials move by the magnetic force and/or the torque as a function of a dipole moment that varies from material to material without power dissipation. The magnetic force is directly proportional to the field gradient, which pushes the materials to the local maximum field. The torque is proportional to the external field that aligns the magnetization of the particle along the field [13].

The applications of an external magnetic field have been studied over the long term in different disciplines for broad applications to manipulate the magnetic helical micro-machines [14], control the motion of small-sized particles for the magnetic separation [15], transport the microdroplet on an open surface [16], assist in the water purification [17], enhance the uptake of magnetic nanoparticles via cultured cells [18], evolve gases [19,20], apply specifically in biomaterials [21], operate the motion of smaller-sized particles in solution [22], and control the speed and direction of swimming devices [13]. 

Different techniques of opened- and/or closed-loop systems have been developed in the motion control system [13], along with types of fields used to generate the torque responsible for rotation. Some of them have used a rotating low magnetic field strength [14,23] and magnetic field gradients [24] as well as oscillating magnetic fields [25]. In all these applications and control systems, magnetic particles suspended in a fluid are governed by the magnetic field that is inspired by the magnetic force generated by the magnetic field gradient, the drag, the buoyancy, and the gravitation. The magnetic rotational effect causes their magnetic moments to align in the same direction as that of an applied external magnetic field. 

Herein, the phenomena that happened on magnetite-encapsulated graphene (magnetic graphene) composites [7] are reported and elucidated based on the translational and self-rotational movements guided via an external magnetic field, which are generated by two pairs of electromagnets by employing a feedback control system. The pulse width modulation is proposed as a technique to regulate the electric power supplied into the electromagnet by altering a duty cycle (the proportion of signal-switched ‘on time’ (pulse width) to a total period). Parameters necessary to enhance the output field strength are assessed. The magnetic graphene composites have been prepared by thermally treating the amine-exfoliated stage-1 FeCl_3_-graphite intercalation compound [7,9]. The produced graphite heterogeneously encloses iron oxide nanoparticles and has indicated the superparamagnetic property. After the cubic graphite intercalation compound was thermally treated, it expanded in one direction. Thus, this expanded magnetic graphene composite should be adequate for examining its movement under the electromagnet. The expanded material that disposes randomly the magnetic nanoparticles should be expected to behave uniquely in the external magnetic field and to be elucidated its behavior. Additionally, the influence of a magnetic nanoparticle to HeLa cells for toxicity investigation under the electromagnet has been examined for widening the usage of the magnetic nanoparticles in the future. 

The first goal of the present study is to elucidate the movement of graphene particles encapsulating magnetic nanoparticles under the magnetic field, which is generated by two pairs of magnets located perpendicularly to each other. The second goal is to notice the impact of the clarified movement on cancer cell growth as biomedical application research. The advantages of using magnetite-embedded graphene particles instead of traditional magnetite particles can be explained based on the pre-examination using magnetite: Pure magnetite nanoparticles do not disperse in water, induce cytotoxicity, and tend to be engulfed by immune cells before they can reach the disease area of the human body. Thus, the biocompatible coating of magnetic particles can avoid such demerits. Magnetite-embedded graphene particles are better suited for the present research on their fundamental behavior under magnets and also on biomedical applications.

## 2. Theoretical Background 

The motion of the magnetic particle at the air–liquid interface can be governed by different forces, such as the magnetic force due to the external magnetic field, the viscous drag force applied on the magnetic particle, the gravitational force, the buoyancy force, the surface tension, the thermal force and the particle–liquid interaction [24]. In most applications that involve nano- to micron-sized particles, the forces due to the magnetic field strength and the drag are more dominant than other forces [26,27]. Thus, it is important to assess some electromagnetic principles and their effects on change in the position of a magnetic particle exposed to the external magnetic field [28].

In the principle of magnetism, a total magnetic field ***B*** (also known as magnetic induction) is a combination of the field from the electric current field ***F*** and the magnetization ***M*** of magnetic materials exposed to the external field according to Equation (1) [29].

***B*** = μ_0_(***F*** + ***M***) (1)

where $\mu_{0}$ = 4π × 10^−7^ TmA^−1^ is an invariant magnetic permeability in the free space. The magnetic torque τ on a magnetic dipole moment ***m*** in a magnetic induction is then simply given by Equation (2)

(2) τ=m × B

This indicates that the magnetic induction will align the magnetic dipole moment (or the current loop) in a way parallel to it. Thus, Equation (2) explains the turning effect of the magnetic graphene because there is no rotational motion without torque. In such a situation, the magnetic energy *E_m_* can be given in Equation (3) [22];

*E_m_* = −[v/(2μ_0_)]χ***B***^2^ (3)

where v and χ are the volume and the susceptibility of a magnetic particle, respectively. This equation implies that the energy decreases with the magnetic field on a magnetic particle, which is being focused on in this study. Based on the relation between force and energy, the magnetic force ***F_m_*** can be expressed as Equation (4) [22];

***F_m_*** = [(χ_par_ − χ_sol_)/μ_0_]v(***B***·***∇***)***B*** (4)

where $\chi_{par}$ and $\chi_{sol}$ are the susceptibilities of superparamagnetic particle and solution, respectively, and ***B***·***∇*** is the magnetic field gradient. That is, Equation (3) relates the magnetic energy and magnetic induction to define the magnetic force described in Equation (4). In a diamagnetic solution like in water of $\chi_{sol}<0$, the superparamagnetic particle with the positive susceptibility is attracted towards the strong field region (high field seekers), while diamagnetic materials are low field seekers. This is the main difference between superparamagnetic and diamagnetic materials. In solution, one has to account for the magnetic susceptibility of the solvent as well. In this case, the difference in magnetic susceptibility between the solvent and the particle determines the magnetic force. It is followed from Equation (4) that the force on a diamagnetic object can be enhanced by placing it in a paramagnetic solution. Meanwhile, it is necessary to mention that the magnetic force exerted on the magnetic particle depends on the volume of the magnetic particle, its magnetic susceptibility, and the non-uniformity of both applied field and field gradient.

The manipulation of the magnetic materials can be achieved using either homogenous or inhomogeneous external magnetic fields. Although a homogenous external magnetic field has been used frequently [24], it does not affect the magnetic particle [30]. Thus, gradient fields take advantage of producing the translational force on the particle that is able to exert an attractive magnetic force in the increasing intensity of the magnetic field generated with electromagnets equipped with the feedback control system.

Yamaguchi et al. [31] have pointed out mechanisms that allow the orientation of magnetic particles in an external field. The orientation generates an intrinsic anisotropy on the susceptibility of the relevant material that differs between axis and shape anisotropy. As far as the axes are concerned, the simplest case can be a uniaxial material with a difference in susceptibility ∆χ between two main axes. When an angle θ between the axis of the lowest magnetic susceptibility and the external magnetic field is considered, the magnetic energy can have a form of Equation (5) [22,32];

*E_m_* = −[v/(2μ_0_)]Δχ(***B***cosθ)^2^ (5)

Thus, the magnetic material will orient itself with an axis related to the highest magnetic susceptibility that is parallel to the external magnetic field. Additionally, the shape anisotropy is due to the so-called demagnetization effect, and the induced magnetic dipole moment is also shape-dependent. Thus, the longer axis of the magnetic material rather than its shorter axis advantageously aligns parallel to the applied magnetic field and reduces the induced demagnetizing field that results from the surface magnet charges. That is, the alignment along the short axis results in the enhanced demagnetizing field and is, therefore, energetically unfavorable. 

## 3. Experimental Methods 

### 3.1. Materials

Thermally expanded magnetic graphene composite particles are the same sample as previously synthesized and characterized [7]. As reported, the amine-exfoliated stage-1 FeCl_3_-graphite intercalation compound was thermally treated 2 min at 900 °C. The superparamagnetic product named G-900 had a mass-susceptibility of 1.256 × 10^−5^ m^3^kg^−1^ and a saturated magnetization of 2.5 Am^2^kg^−1^. Particles with different sizes and shapes from the as-synthesized particles were supplied to the experiment. Selected particles were short, elongated, and curved particles, and they have shorter axis lengths and longer axis contour lengths of 0.2 and 0.8, 0.3 and 1.6, and 0.3 and 2.0 mm, respectively. Cooking oil, honey, albumin, egg yolk, and jellies are household food products. 

### 3.2. Setup of the Apparatus

**Figure 1:**
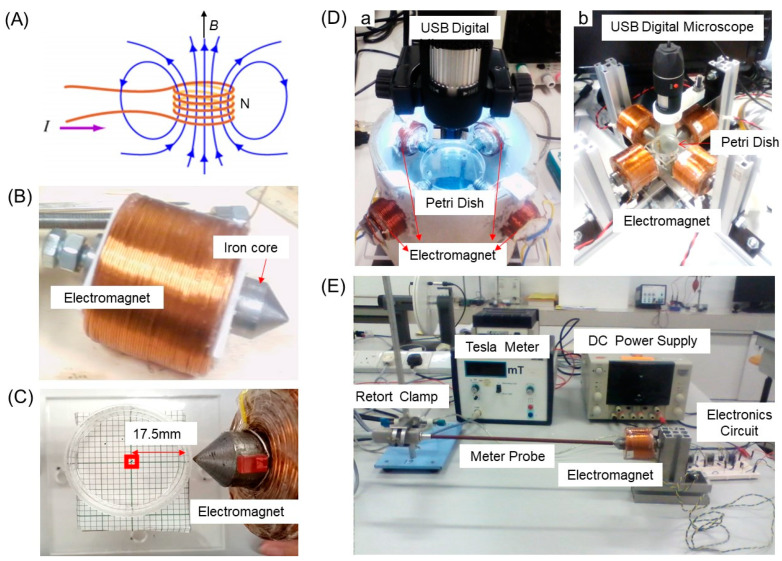
(**A**) A schematic representation of a current-induced magnetic field on copper coil, (**B**) an electromagnet built with a soft iron core which was designed to focus and provide maximum magnetic field at the tip, and (**C**) the location of a magnetic particle from a soft iron tip. (**D**) A prototype equipped with electromagnets of (**a**) 500 and (**b**) 1000 turns and (**E**) an experimental setup where a magnetic field density *B* generated from a constructed electromagnet is measured using a Tesla meter.

A solenoid made of copper wire winded in a tight helical shape was used as a device to generate a homogeneous magnetic field (field strength invariant in space and time). As shown in Figure 1A, the field *B* generated was uniformly directed parallel to the solenoid axis via passing an electric current *I* through N turns of copper wire and could be determined by summing fields generated by the individual turn of wire. The length of a solenoid was greater than the diameter of a tightly winded coil, whose translational and rotational symmetry was considered. An electromagnet, shown in Figure 1B, essentially consists of an iron core bent at its end, which was designed to enhance the field strength by means of the current through a coil. 

Four electromagnets were symmetrically arranged in four directions, and the end of iron core of each electromagnet was put in touch with a petri dish of 35 mm in diameter and 10 mm in height (Figure 1C), which was set in the center of four electromagnets (see Figure 1D) and filled with water. In comparison, different types of liquids (cooking oil, honey, albumen, egg yolk, jelly (less water and more water), and bacteria-cultured medium) were used. Images of magnetic particles in a petri dish and an image of the calibrated ruler (a grid with defined dimension) under the same magnification were captured with a USB microscope (50X-500X 8-LED, AmScope, Irvine, CA 92614, USA) set on the petri dish. The resolution of the camera was 30 fps with the workspace. These images were converted to pixels using MATLAB R201 3b software (Mathworks, Natick, MA, USA).

The whole setup for the observation of particle motion under the applied external magnetic field is shown in Figure 1E. A Tesla meter (PHYWE, Gettingen 1037079, Germany) was fixed to measure the magnetic field generated, and it was calibrated up to 1 T. Two pairs of electromagnets, each of which was connected to an electronic switch and used to influence a relevant magnetic particle, are perpendicular to each other in a way to control the motion of a particle placed in the workspace. The space between the electromagnets, as well as the motion of a relevant particle, was recorded and imported to a computer by a USB (50X-500X 8-LED) digital camera mounted at 90° for the surface of a liquid. 

### 3.3. Cell Growths and Cell Viability Assays 

The test was performed for rotating graphene nanoparticles (at concentrations of 100, 500, and 1000 μg/mL in solution) onto the HeLa cell lines, and different durations of magnetic moments (2, 3, 7, and 10 min) were applied. Three sets of experiments were prepared: The first set was a controlled experimental treatment (Experiment 1) to investigate the effects of normal atmospheric conditions on HeLa cells without any testing for cytotoxicity effect of the particles onto the cells. Experiment 2 was carried out for the different concentrations of short magnetic graphene nanoparticles without rotational movement. In the last experiment (Experiment 3), the different durations of rotational magnetic moment were applied to the cell lines. 

The trypan blue exclusion assay was used to assess the viability of cancer cells in the laboratory. In this assay, cancer cells were treated with trypan blue, a dye that cannot penetrate the intact cell membranes of healthy, viable cells. Viable cells exclude this dye, appearing clear under a microscope, while non-viable cells with compromised membranes allow the dye to enter and stain them, causing them to appear blue. Then, by counting both stained (non-viable) and unstained (viable) cells, the percentage of viable cells was calculated.

## 4. Results and Discussion 

### 4.1. Parameters of Motion Control System and Instrument 

**Figure 2:**
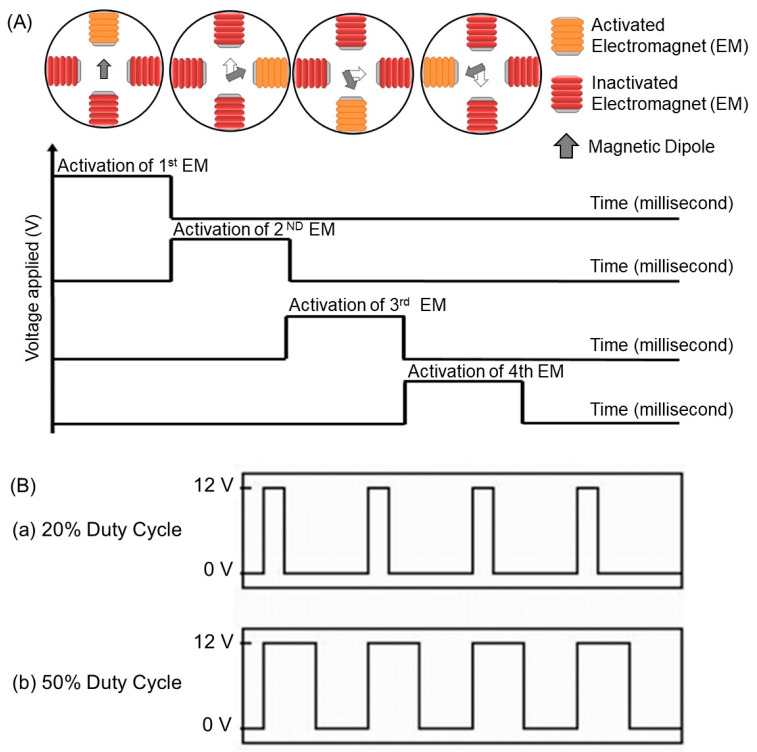
(**A**) Basic concepts for moving a magnetic particle. (**B**) Switching a signal modulated to alter the electrical current supplied to electromagnet by (**a**) 20% and (**b**) 50% duty cycles.

Magnetic graphene composites of different sizes and shapes were placed at the center of four electromagnets in the OFF state on the air–liquid interface in a petri dish, and for the relevant particle to move in two dimensions under the applied magnetic field, algorithms were set to generate a dynamic field which consequently applies a torque on the particle. As seen in Figure 1E, each electromagnet was connected to a power transistor (an electronic switch) that switches ON the electromagnet by letting an electric current flow into it from a 24 V power supply. A microcontroller was programmed to switch ON/OFF of the transistor in a fast manner. Here, four electromagnets were consecutively switched ON and OFF. The particle was at first attracted to the first activated electromagnet, followed by the second, the third, and then the fourth one, as shown in Figure 2A, and the process repeatedly turned back to the first one (named each activated one from the electromagnet 1 (EM1) to electromagnet 4 (EM4), consecutively). Each electromagnet took 10 ms for its turn to produce the magnetic field. 

Pulse width modulation (PWM) can be employed to regulate the power supplied into the electromagnet by altering a duty cycle to deliver an electric current. By modulating this pulse width, the voltage and, thus, the electric current into the electromagnet can be changed. As shown in Figure 2B, the bigger the pulse width of the 50% duty cycle, the more electric current is supplied, and hence, the greater magnetic field is yielded as compared with that of the 20% duty cycle.

**Figure 3:**
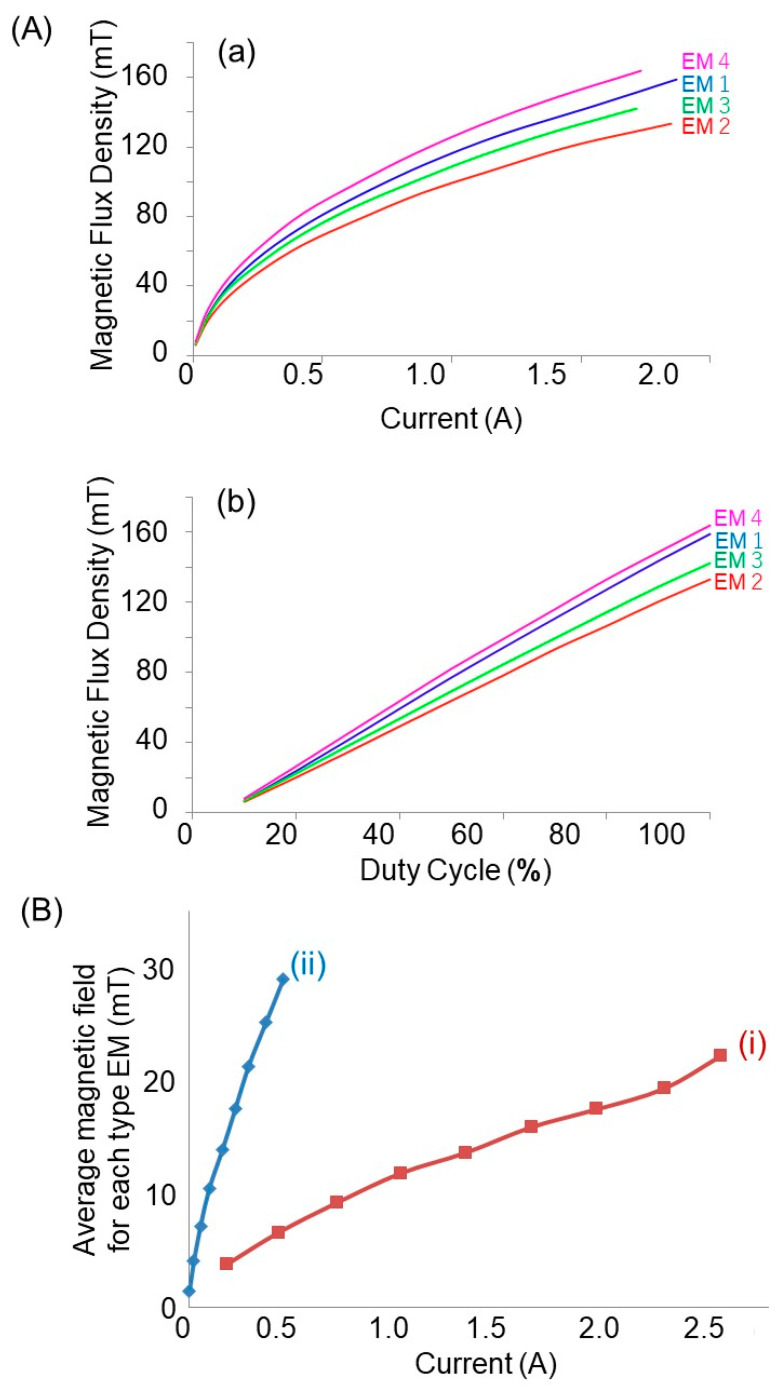
(**A**) A magnetic flux density as a function of (**a**) electric current and (**b**) continuous duty cycle. (**B**) An average rotating magnetic field as a function of current draw for winding electromagnet at (i) 500 and (ii) 1000 turns. The field was measured with the Tesla meter. EMx(x = 1–4) means electromagnet 1–4.

The magnetic field generated by electromagnets has a magnitude equal to *B* = µ_o_µ_r_ *I* n, where u_r_ is the relative permeability of the iron core, and n is the number of turns per unit length of the solenoid coil. Based on this equation, the possibility of enhancing the field intensity can be identified as increasing the current passing through the coil. Figure 3(Aa) draws the influences of a current on the magnetic flux density. The non-linear behavior is due to the magnetic core. The higher current *I* drawn from the power supply will end up creating more heat (power dissipation I^2^R where R is the resistance) on the electromagnet, which lessens the efficiency in generating a magnetic field. Figure 3(Ab) indicates that the most important possibility is a route used to enhance the intensity based on the duty cycle ratio, and the larger the duty cycle, the stronger the intensity supplied. From these results, it can be found that the duty cycle and the magnetic field intensity are in a linear relationship. Then, Figure 3A indicates that the magnetic flux density has a quadratic relation with the electric current provided and a linear relation with the continuous duty cycle. 

Figure 3B plots the magnetic field as a function of the current draw for electromagnets winding 500 and 1000 turns. Increasing the number of turns increases the magnetic field intensity, even at a small amount of current drawn to the electromagnet. These analyses (Figure 3) show that the magnetic flux density generated is dependent on the number of coil turns, the electric current draw, and the continuous duty cycle.

### 4.2. Movement of Magnetic Graphene Particles 

**Figure 4:**
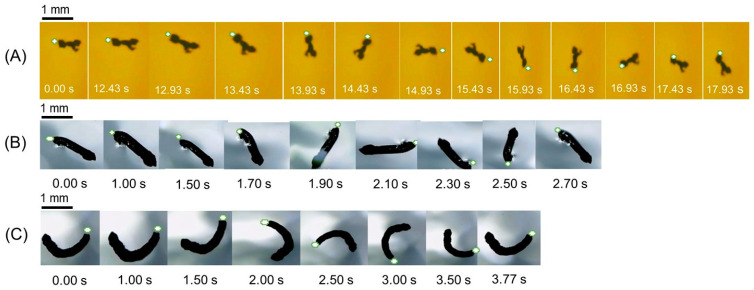
Path follows snapshots of (**A**) short, (**B**) elongated, and (**C**) curved magnetic graphene particles at different time intervals.

Magnetic graphene composite particles, where magnetite nanoparticles are dispersed in graphene with short, elongated, and curved structures, were placed on the air–water interface, and their movement behaviors were observed under the successive algorithm of four electromagnets programmed to switch ON/OFF. The videos were recorded from the initial location of the magnetic particle (the center of electromagnets) to its initial translational movement for some fixed duration, following the autorotational movement at any distance from a center. On the autorotation, the relevant particle rotated on the axis through the center of gravity in a particle. Videos were named Videos S1–S3 for short, elongated, and curved magnetic graphene particles, respectively, and movement paths followed by each magnetic particle were analyzed from the video snapshot and shown in Figure 4 at some fixed time interval. To follow the orientation and the direction of rotation, the image of the particle was marked at one end. The translational motion of the elongated particle was recorded in Video S4.

**Figure 5:**
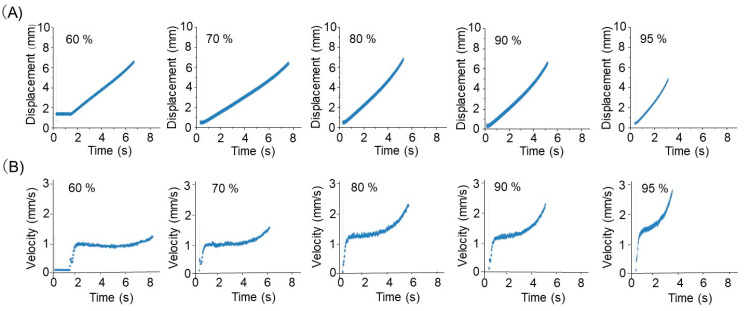
(**A**) Displacement–time and (**B**) velocity–time graphs of the translational movement of a short magnetic graphene particle at different duty cycles. The percentage values in the figures indicate the duty cycle.

Video S1 and Figure 4A indicate the concurrent translational and rotational motions of short magnetic particles at the air–water interface. That is, the particle transferred its center of gravity with rotation after 12.43 s (see Video S1 and Figure 4A). Figure 5 plots the displacement versus time and the velocity versus time graphs of the translational movement of short particles collected at different (60–95%) duty cycles from Video S4. As seen in a displacement–time graph (Figure 5A), at a 60% duty cycle, the particle was not moving till 1.4 s, in which the electromagnet of the system was not activated yet. However, the particle started to move as early as below 0.5 s at higher duty cycles. The displacement increased almost linearly to the time, and the speed of ~1 mm/s at 70% duty cycle was hastened at a higher duty cycle. These aspects are apparent from the velocity–time profile in Figure 5B. The induced field by the electric power supply made the short particle move at an almost constant velocity, like 1.0–1.3 mms^−1^, at the low-duty cycles after the initial increase. However, a further increase in velocity was observed at the high-duty cycles. It was about 1.5 mm/s at 95% duty cycle.

**Figure 6:**
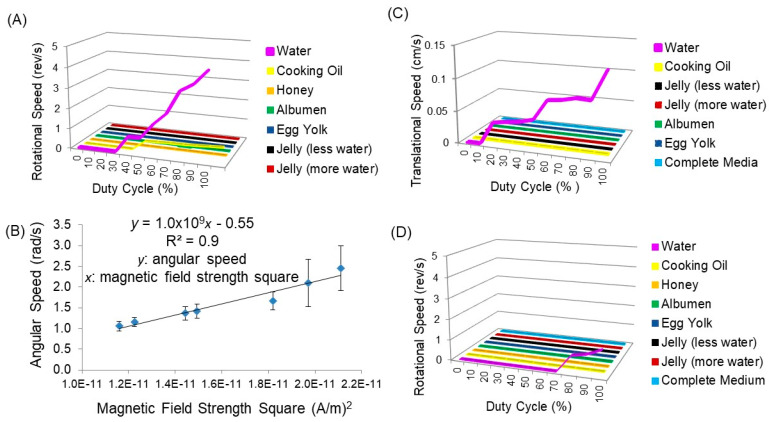
(**A**) Rotational speed as a function of duty cycle and (**B**) average angular speed versus magnetic field strength square of short magnetic graphene particle, and (**C**) translational speed and (**D**) rotational speed as a function of duty cycle of elongated magnetic graphene particle in different fluidic media operated for 40 s at 24 V. Angular speed of particle was calculated using a formula 2π/T, where T is the time taken for one point of the particle to complete one rotation. Rotational speed focuses on the number of full rotations made by an object in a given time, whereas angular speed concentrates on the rate of change of angular position or the angle swept out per unit of time. They are related by the formula: Angular speed (radian) = 2π × Rotational speed.

Meanwhile, the rotational speed of a short particle versus duty cycle at the air–water interface plotted in Figure 6A was not observed at an increased duty cycle of up to 30%. The speed increased almost linearly with the increase of duty cycle after 30% and reached 4.5 rev/s at 100% duty cycle. Although just the rotation was observed even at the air-cooking oil interface, the speed was constant above 40% duty cycle. Incidentally, movements did not occur on arbitrarily selected other liquids. As shown in Figure 6B, the angular speed of a short magnetic particle was dependent on the magnetic field strength; it linearly increased for the field strength square at 1.2 × 10^9^~2.2 × 10^9^ (A/m)^2^. This result creates the empirical rule of angular speed ω = k*B*^2^ + constant. 

It was observed from Video S2 and Figure 4B that after the 1.00 s induction of electromagnets, an elongated particle started the rotational motion and took about 1.70 s (1.00–2.70 s) to complete one cycle. Its translational motion in Video S4 apparently continued 7.70 s after the system was activated. Figure 6C,D also displayed translational and rotational speeds as a function of a duty cycle for an elongated particle compared at different fluid surfaces. The particle revealed both translational and rotational movements only on the less viscous water surface for the operation of 40 s at a 24 V power supply. The translational speed of an elongated particle increased at the duty cycles above 10% to 1.2 mm/s at 95% duty cycle (Figure 6C). Meanwhile, the rotational speed started to record from a 70% duty cycle and reached 1.0 rev/s at a 100% duty cycle (Figure 6D). Both translational and rotational motions of an elongated particle at the air–water interface increased with increasing the duty cycle above the threshold value described above. Results confirmed the effectiveness of the high-duty cycle to obtain the high rotational speed of an elongated particle. Moreover, the comparison of speed indicates the faster translational and rotational movements of a short particle than an elongated particle.

A curved particle simply autorotated at the original position and did not translationally move when electromagnets were applied (Video S3, Figure 4C). That is, instead of being pulled toward the electromagnet (the translational movement), a curved particle autorotated in response to the fast switching of four electromagnets. Then, the rotation took 2.77 s (1.00–3.77 s) per cycle (Figure 4C). Since this time is longer than that (1.70 s) of an elongated particle, the rotational speed of a curved particle is slower than that of an elongated particle. 

### 4.3. Mechanisms of the Rotational Motion of Magnetic Graphene Particles

A torque has been applied to magnetic graphene particles to enable the axis spinning by means of four electromagnets with a feedback control system. The system generates magnetic forces onto particles and hence pulls particles to change their position (Figure 4). A magnetic particle of micrometer size in the transport system at the air–liquid interface is influenced by different types of forces. When a small-sized particle moves on a (gravitational) fluid, the buoyant force is too small. The force that depends on the nature of the fluid should be controversial; it may be a drag force, *F*_d_, where the particle motion depends on the viscosity of the fluid. When the particle is small in size and moves slowly at the air–liquid interface, its drag force is proportional to its velocity, as given by Stokes’ law, *F*_d_ = −6πrηv [27,28], where r is the radius of the particle, η is the viscosity of the fluid, and v is the velocity of the particle. The viscosity of fluid (e.g., 1.00, 44.7, and 6426 mPa·s for water, cooking oil, and honey, respectively) involves the rotational speed of a particle for all the analyzed fluid media. Thus, the drag force is lower at the air–water interface than at the surface of other liquids examined here.

When molecules in the bulk liquid exert a force on one another in all directions, the net force becomes null due to only the cohesive force. Meanwhile, at the surface of the liquid, the unbalanced inward force generates an internal pressure, namely, the surface tension that forces the liquid surface to shrink as a result of the adhesive nature of the force between the air in the upper phase and liquid molecules in the lower phase. Due to the unbalanced inward force, the surface tension is higher at the air–water interface than at the surface of other liquids examined here.

#### 4.3.1. A Short Magnetic Graphene Particle

**Figure 7:**
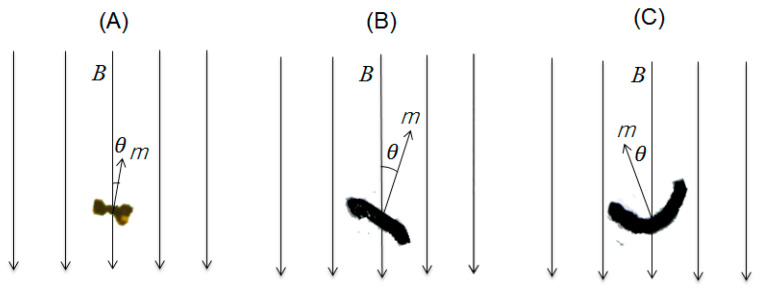
The initial orientation of (**A**) short, (**B**) elongated, and (**C**) curved magnetic graphene particles in the external magnetic field system. B is the applied field, m is the magnetic moment vector, and θ is the angle of particle rotation.

In general, larger particles experience a large magnitude of drag forces, and smaller ones suffer a stronger fluid ordering. A short magnetic graphene particle showed more positional change and faster movement at the air–water interface as well as on the oil fluid medium surface than both elongated and curved magnetic particles because a fully-small short particle satisfies conditions of the low drag force on the surface of low viscosity fluids and the fast ordering on the external magnetic field. It also can orient itself with an axis related to the high magnetic susceptibility for the external magnetic field (see Equation (5)) (Figure 7A). 

#### 4.3.2. An Elongated Magnetic Graphene Particle

It was identified that the rotational movement of an elongated magnetic graphene particle was initially observed after 1.0 s. Until this time, the condition may be insufficient to influence the rotation of a particle due to the drag force originating from the nature of the liquid. After the magnetic force dominated the drag force, a magnetic particle started to rotate on its own axis. An elongated magnetic particle starts the rotation at a 70% duty cycle, whereas a short particle rotates above a 30% duty cycle at the air–water interface. Moreover, the movement of the former is slower than the latter. These differences originate from the size difference between two particles: an elongated particle is heavier in weight than a short particle. The heavier the weight, the higher the moment of inertia (the resistive drag force). Then, an elongated particle needs a stronger magnetic field strength (the higher duty cycle) than a short particle. Meanwhile, an elongated particle rotated in a clockwise direction, as well as a short particle. The direction of the rotation may be affected by its initial orientation with respect to the external magnetic field at the air–water interface (Figure 7B). 

#### 4.3.3. A Curved Magnetic Graphene Particle

After the dominance of the magnetic force, the rotation of a curved magnetic particle started after 1.00 s. Until 1.00 s, the condition may not be enough to influence the motion of a particle due to the resistive drag force. Without the translational movement, a curved magnetic particle rotated on its own axis in a counterclockwise direction. The rotation is affected by the initial orientation of a particle with respect to the external magnetic field at the air–water interface (Figure 7C). This is the same reason applied to the direction of rotation of short and elongated particles as described above. Moreover, the direction of the rotation may be influenced even by the surface tension around the curved shape [33]. A curved particle is nonlinear in shape, different from both the short and elongated particles. In addition, since the surface magnetic charges are induced on its surface, the orientation of a curved particle against the external field may have a higher demagnetization effect than an elongated one. Thus, the rotational movement of a curved particle is slower than that of an elongated particle and rather restrained.

### 4.4. Effects of Magnetic Graphene Nanoparticles on Cell Growth 

**Figure 8:**
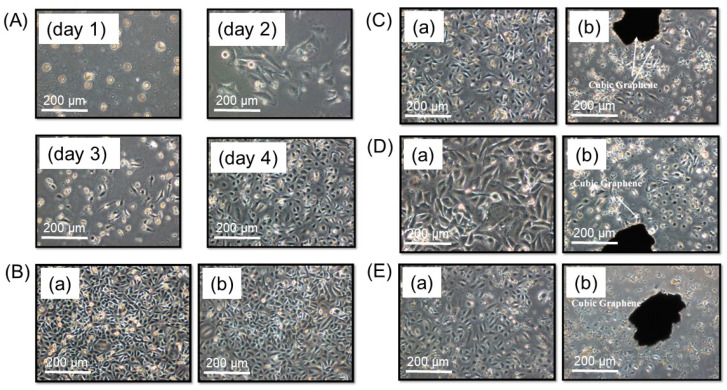
(**A**) Morphology of HeLa cell growth monitored for up to 4 days on a culture dish without magnetic particles. (**B**) Morphology of HeLa cells (**a**) before and (**b**) after 10 min rest with no further treatment in the control experiment without magnetic particles. Morphology of HeLa cells soaked in (**C**) 100 μg/mL, (**D**) 500 μg/mL, and (**E**) 1000 μg/mL short graphene nanoparticles (**a**) before and (**b**) after 10 min exposure under applied magnetic field from the prototype.

Figure 8A shows the morphological variation of cells during the cell growth up to 4 days. As seen from the figures, 70% or more cells increased daily. Images of HeLa cells as a control batch with no applied treatment for a duration of 10 min rest time in Figure 8B exhibited no obvious changes in cell morphology. Meanwhile, Figure 8C–E display the morphology of HeLa cells at different concentrations (100, 500, and 1000 μg/mL, respectively), in which all received 10 min rotation of magnetic graphene by means of the applied magnetic field, being different from the prototype. The population of HeLa cells decreased, as seen in Figure 8C. A greater reduction of cells was indicated in Figure 8D,E. The finding here indicates that a greater magnetic graphene concentration on the cell line depresses cell growth. It might be necessary to identify the part of the composite with the biological effect, and this will be performed in further investigations through any theoretical support [34,35].

**Figure 9:**
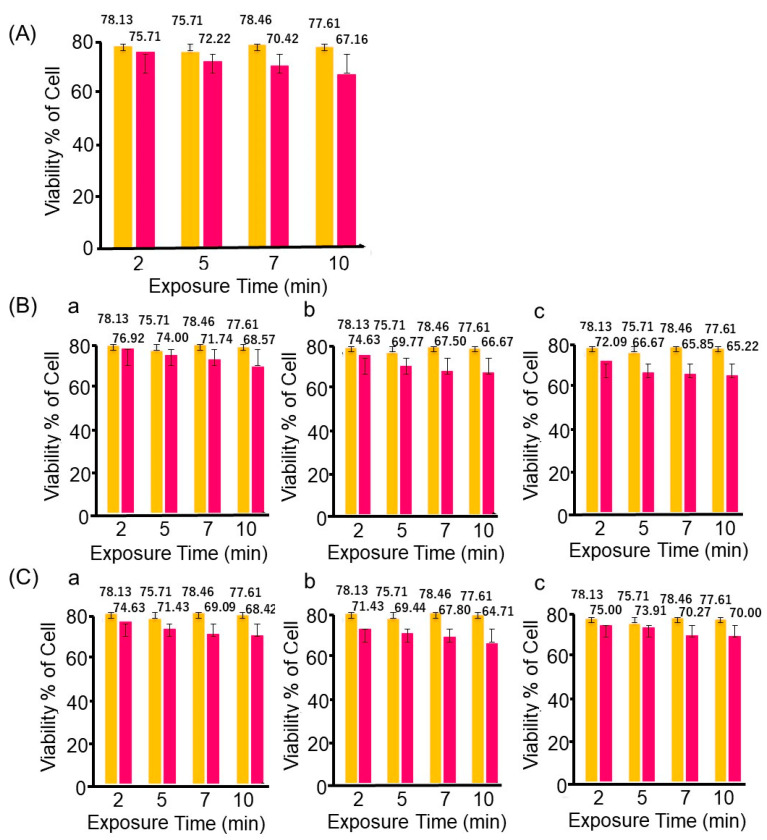
Plots of percentage cell viability versus exposure duration in (**A**) Experiment 1 (without short magnetic graphene and rotation), (**B**) Experiment 2 (without rotation), and (**C**) Experiment 3 (with rotation). In (**B**,**C**), (**a**) 100; (**b**) 500; (**c**) 1000 μg/mL of short magnetic graphene. Orange: reference, red: sample.

For the cell viability assay, cells were marked by a trypan blue solution to measure the total number of healthy and dead cells. HeLa cells were merely exposed without treatment to the magnetic graphene for a duration of 2, 5, 7, and 10 min in Experiment 1. In studies of Experiments 2 and 3, HeLa cells were loaded with short graphene nanoparticles at different concentrations such as 100, 500, and 1000 μg/mL for 2, 5, 7, and 10 min on investigation without and with applied external magnetic field. All results are presented as a bar chart in Figure 9 compared to reference data free from HeLa cells, magnetic nanoparticles, and magnetic field.

The result in Figure 9A indicates that the viability of HeLa cells decreased in the extended exposure to the atmospheric condition in Experiment 1. Similarly, the same phenomenon on the viability of the HeLa cells occurred along with the increase in exposure time as the concentration of the magnetic particles increased, as shown in Figure 9B. Hypothetically, the higher concentration (1000 μg/mL) of the magnetic graphene is expected to increase the cell viability as compared to the lower concentration (100 and 500 μg/mL) of magnetic graphene-containing cells, but this efficiency was less (see in Figure 9B). Meanwhile, according to the results of Experiment 3, as shown in Figure 9C, the most death of cells occurred at 500 μg/mL dispersion than at 1000 μg/mL dispersion. One of the possible reasons might be due to the self-assembling of graphene nanoparticles. Although magnetic particles at 500 μg/mL are able to rotate among the cells, the agglomerates at 1000 μg/mL induced by the electromagnetic field might hardly rotate or move around the cell lines, and therefore, this results in a lower cell inactivation as an outcome. The differences in cell viability at 10 min exposure time are 65.2–68.5% and 64.7–70.0% in Experiments 2 (without magnetic field) and 3 (under magnetic field), respectively. This result indicates that the rotational effect of the graphene particle is less active for the viability of HeLa cells. 

The translational and rotational movements of graphene particles generate mechanical forces on the cellular membrane, initiating mechano-transduction pathways within cells that can lead to alterations in gene expression, cell signaling, and behavior [36]. These mechano-sensitive responses can profoundly impact cellular growth, differentiation, and ability to adapt to environmental cues. Furthermore, translational motion can facilitate the cellular uptake of graphene particles, allowing them to engage with intracellular structures and processes [37]. Depending on their size, shape, and surface properties, internalized magnetic graphene particles have the potential to influence organelles, cellular metabolism, and gene expression, thus exerting a substantial influence on cellular growth and function. Moreover, the direct physical interaction between moving graphene particles and cell membranes triggers various signaling pathways, encompassing those related to cell adhesion, inflammation, and stress responses [38]. These intricate cell-particle interactions play a pivotal role in shaping cellular growth and behavior, making them integral components of focus in the present study on the multifaceted impact of dynamics of magnetic graphene particles.

## 5. Conclusions 

In this study, the system that adducts the external magnetic field to the air–liquid interface was prepared to evaluate the principal movement of magnetic particles and its effect on the HeLa cells. Magnetic graphene particles were remotely controlled by applying the external magnetic field. The short particle suffered a faster motional effect than other magnetic particles examined. From the motion of the short particle, a quadratic relation was found for the angular speed versus magnetic field parameters. Next to the short particle, the elongated magnetic graphene particle could successfully rotate at an angular speed lower than that of the short particle, whereas the curved particle rotated at the slowest speed in the same duty cycle employed to the system on the less viscous medium (here water). These results indicate that the duty cycle proposed is suitable for rotating micro-sized particles, and the rotational speed is also size/shape-dependent. The mechanism proposed also supports the fact that the only short particle has shown the motional effect even on an oily fluid medium. From the results, one can conclude that the higher the viscosity of the fluid, the greater the influence of the drag force. That is, the less viscous fluid, here water, the lower the effect of the drag force and hence the higher the dominant magnetic force and vice versa. Since this system can operate the movement of the magnetic particle by the remote control, it will be a non-invasive tool not only for the essential movement of magnetic particles but also for biomedical purposes like cell growth induced by a dynamic magnetic field. The practical application significantly showed that the growth of cancer cell lines drops with rotational movement under the addition of magnetic graphene. Thus, the present system using magnetic fields will contribute to the fresh advancement of biomedical research alongside the development of lab-on-chip applications of rotational magnetic particles [39].

## Data Availability

Not applicable.

## Supplementary Materials

Supplementary data to this article can be found online at https://www.mdpi.com/article/10.3390/nano13202806/s1. Video S1 of movement of short magnetic graphene particle. Video S2 of movement of elongated magnetic graphene particle. Video S3 of movement of curved magnetic graphene particle. Video S4 of translational movement of elongated magnetic graphene particle.
